# The pace and prognosis of peripheral sensory loss in advanced age: association with gait speed and falls

**DOI:** 10.1186/s12877-018-0970-5

**Published:** 2018-11-12

**Authors:** Lewis A. Lipsitz, Brad Manor, Daniel Habtemariam, Ikechukwu Iloputaife, Junhong Zhou, Thomas G. Travison

**Affiliations:** 1 Hebrew SeniorLife Institute for Aging Research, 1200 Centre Street, Boston Roslindale, MA 02131 USA; 2 Division of Gerontology, Beth Israel Deaconess Medical Center, Boston, MA USA; 3 Harvard Medical School, Boston, MA USA

**Keywords:** Neuropathy, Elderly, Mobility, Longitudinal, Feet

## Abstract

**Background:**

Peripheral sensory loss is considered one of many risk factors for gait impairments and falls in older adults, yet no prospective studies have examined changes in touch sensation in the foot over time and their relationship to mobility and falls. Therefore, we aimed to determine the prevalence and progression of peripheral sensory deficits in the feet of older adults, and whether sensory changes are associated with the slowing of gait and development of falls over 5 years.

**Methods:**

Using baseline, and 18 and 60 month followup data from the **M**aintenance **O**f **B**alance, **I**ndependent **L**iving, **I**ntellect, and **Z**est in the **E**lderly (MOBILIZE) Study in Boston, MA, we determined changes in the ability to detect stimulation of the great toe with Semmes Weinstein monofilaments in 351 older adults. We used covariate-adjusted repeated measures analysis of variance to determine relationships between sensory changes and gait speed or fall rates.

**Results:**

Subjects whose sensory function was consistently impaired over 5 years had a significantly steeper decline in gait speed (− 0.23 m/s; 95% CI: -0.28 to − 0.18) compared to those with consistently intact sensory function (− 0.12 m/s; 95% CI: -0.15 to − 0.08) and those progressing from intact to impaired sensory function (− 0.13 m/s; − 0.16 to − 0.10). Compared to subjects with consistently intact sensation, those whose sensory function progressed to impairment during followup had the greatest risk of falls (adjusted risk ratio = 1.57 (95% confidence interval = 1.12 to 2.22).

**Conclusions:**

Our longitudinal results indicate that a progressive decline in peripheral touch sensation is a risk factor for mobility impairment and falls in older adults.

## Background

Falls are a leading cause of morbidity and mortality among older adults, occurring in approximately one-third of community-dwelling persons over age 65 and costing over $31 billion in related injuries annually [1–3]. The presence of peripheral sensory loss is considered to be one of many different risk factors for falls [3–8]. A few previous studies demonstrated that older adults with peripheral sensory loss at the outset had an increased risk of subsequent falls [5–9]. However, these studies relied on single baseline measures of sensory function and did not examine the effect of changes over time [7, 8]. The longitudinal relationship between changes in peripheral sensory function and the development of falls is not known.

Peripheral neuropathy is common among older adults, [10] especially in those with diabetes [11, 12]. The prevalence of peripheral neuropathy has been reported to be approximately 7% in older adult populations worldwide [11, 13]. While there are many causes, including alcohol ingestion, vitamin B_12_ deficiency, cancer, chemotherapy, chronic kidney disease, and paraproteinemias, diabetes is the most frequent, accounting for 32–44% of patients with polyneuropathy [14, 15].

Many factors associated with peripheral neuropathy may increase the risk of falls, including impaired balance, muscle weakness, nutritional deficiencies, and medications [2, 16]. One mechanism by which the progression of sensory neuropathy may predispose people to falls is through alterations in gait. While slow gait speed has been reported to be cross-sectionally associated with distal sensory neuropathy [17], this could be either a cause of falls or protective mechanism to prevent falls. The present study takes advantage of longitudinal data from the **M**aintenance **O**f **B**alance, **I**ndependent **L**iving, **I**ntellect, and **Z**est in the **E**lderly (MOBILIZE) Study in Boston, MA, to assess the prevalence and progression of peripheral sensory deficits in the feet, and whether they are associated with slowing of gait and falls over a 5-year period.

## Methods

### Participants

The MOBILIZE Boston Study (MBS) is a prospective cohort study of a unique set of risk factors for falls in community-dwelling seniors living in the Boston area. The design and methodology for this study have been previously described in detail [18, 19]. In brief, 765 persons eligible for the MBS were enrolled using door-to-door population based recruitment. To be included, individuals had to be: > 70 years of age (or > 65 years if living with a participant), able to understand and communicate in English, able to walk 20 ft without personal assistance (walking aids permitted), expecting to live in the area for at least 2 years, and able to provide written informed consent. Exclusion criteria included terminal disease, severe vision or hearing deficits, and a Mini-Mental State Examination score < 18 [20, 21].

All subjects underwent a complete home and laboratory assessment of demographic characteristics, medical conditions, medications, functional status, gait speed, smoking status, alcohol use, blood pressure, and cerebral hemodynamics, then were followed prospectively for falls using a monthly postcard calendar (see below). These assessments were repeated at 18 months and 5 years, while monthly falls data were collected continuously.

The analysis described here utilized longitudinal data from three waves of MBS data collection over approximately five years. Of the original 765 MOBILIZE Boston participants, data from only 351 subjects were available for the current study because of death, institutionalization, or loss to followup. As expected, the study sample that survived the 5 year followup was healthier that the original cohort, with better scores on the Mini-mental State Examination [20], Trail-making Test [22], Short Physical Performance Battery [23], and Berg Balance Scale [24], and a lower prevalence of diabetes, less use of a walking aid, and faster gait speed.

### Clinical measures

#### Sensory function

We used an abbreviated Semmes-Weinstein monofilament test (SWMT) to assess the threshold for light touch sensation on the dorsum of each great toe, which uses a buckling monofilament to impart a known force to the skin, following the procotol suggested by Perkins et al. [25] The dorsum of the toe was used to avoid callouses on the plantar surface, which interfere with the sensory stimulus. The SWMT is a diagnostic tool to evaluate loss of protective sensation that often leads to ulcer formation. We employed two of the most-widely used monofilaments: the 5.07 monofilament (providing a standardized 10 g buckling force) and the 4.17 monofilament (providing a standardized force of 1.4 g). Failure to feel the 5.07 monofilament represents a loss of protective sensation [26, 27]. The 4.17 monofilament is used to determine whether the participant has normal light touch sensation. Inability to detect the 4.17 may indicate early neuropathy. The protocol began with the 4.17 monofilament, and if the participant could not feel it, he or she was tested with the 5.07 monofilament.

Monofilament testing was conducted while the participant was lying on an exam table, without shoes or socks. The procedure was demonstrated on the participant’s hand or arm before foot measurements were taken. Touch sensation was assessed at a single site on the dorsum of the great toe, 1 cm proximal to the nail bed (distal to the knuckle). The participant covered or closed their eyes throughout the test.

The test results were categorized into sensory loss groupings of intact, mild-to-moderate, and severe deficits, as defined in Table 1. The prevalence of each group is also shown. Changes in sensation over 5 years were grouped into 4 categories as shown in Table 2; including intact to intact, intact to mild-to-moderate impairment, mild-to-moderate impairment to severe, or severe to severe. Nine subjects who exhibited improved sensation were excluded from the longitudinal analysis.

**Table 1:** The definition and prevalence of each category of somatosensory impairment

	Definition	Prevalence at baseline, n (%)
Intact	Able to feel *at least* 3 monofilament touches out of 4 attempts for a 4.17 g monofilament in the left *and* right great toes	292 (83%)
Mild-moderate impairment	Able to feel *fewer* than 3 monofilament touches out of 4 attempts for a 4.17 g monofilament in the left *or* right great toe *and* able to feel *at least* 3 monofilament touches out of 4 attempts for a 5.07 g monofilament in the left *and* right great toes	22 (6%)
Severe impairment	Able to feel *fewer* than 3 monofilament touches out of 4 attempts for *both* 4.17 *and* 5.07 g monofilaments in the left *or* right great toe	37 (11%)

**Table 2:** Categories of change in sensory function over 5 years of followup; count and row percentages shown

Baseline	Follow-up	
	Intact	Impaired
Intact	150 (51%)	142 (49%)
Impaired	9 (15%)^a^	50 (85%)

^a^ *Not included in longitudinal analyses due to small cell size*

#### Gait speed

Gait speed was assessed with a stopwatch as participants walked at their preferred pace over a four meter course. Timing started with a signal while subjects were standing still and ended when they traversed 4 m. To prevent terminal slowing, they were not told where the course ended. They were asked to walk at their comfortable speed as if taking a purposeful walk on the street, going to a store. They were allowed to use an assistive device if they used it at home or outdoors. The fastest time of two separate trials was used for analysis. The time to walk 4 m is a component of the Short Physical Performance Battery (SPPB), described below.

#### Berg balance scale

The Berg Balance Scale is a multi-component assessment of standing balance, consisting of 14 balance tasks with each task scored from 0 to 4, for a summed score of 0 to 56 [24]. The scale has been well-validated and shown to predict risk of falls in community dwelling elders [28]. Only baseline data were available.

#### Physical performance

The Short Physical Performance Battery (SPPB) was used to measure lower extremity mobility performance [23]. The SPPB includes measures of standing balance, 4-m usual-paced walking speed, and ability and time to rise from a chair 5 times. The validity of this scale has been demonstrated by showing a gradient of risk for admission to a nursing home and mortality along the full range of the scale from 0 to 12 [29, 30].

### Falls detection

During a five-year follow-up period, a fall was defined as unintentionally coming to rest on the ground or other lower level, not as a result of a major intrinsic event (e.g., myocardial infarction, stroke, or seizure) or an overwhelming external hazard (e.g., hit by a vehicle) [18, 31]. Participants were instructed to complete and return monthly falls calendar postcards designed to be posted on a refrigerator. On the postcards, participants were to record an “F” for each fall on the day it occurred and an “N” on days when no falls occurred. If the postcard was not returned, a research assistant called the participant to determine whether a fall occurred during the preceeding month and to remind them to complete and return future cards. This approach has been well-validated for use in epidemiological cohort studies and described in previous studies [32]. All subjects who reported falls were also called to determine the circumstances of the fall and clinical outcomes, including whether any injuries (e.g. fractures) and hospital visits occurred.

### Other variables

Sociodemographic characteristics assessed at baseline in the home interview included age, sex, race (self-identified), and years of education. At each wave we used the validated Physical Activity Scale for the Elderly (PASE) to measure self-assessed physical activity in the previous week [33]. Participants were asked about physician-diagnosed major medical conditions.

Diabetes was defined using an algorithm based on self-reported diabetes, use of antidiabetic medications, and laboratory measures from the baseline clinic visit, including random glucose (> 200 mg/dL) and hemoglobin A1C (> 7%). Body mass index (BMI, calculated as weight in kilograms divided by height in meters squared) was calculated from measured height and weight**.** Comorbidity was measured using a count of relevant self-reported medical conditions, including: coronary heart disease, high blood pressure, ulcer or other stomach disease, kidney disease, liver disease, anemia, cancer, depression, osteoarthritis and degenerative arthritis, and rheumatoid arthritis [34]. Baseline subject characteristics also included the Center for Epidemiologic Studies Depression Scale – revised [35], Mini-Mental State Examination and Trail-making Test.

### Data analysis

We summarized baseline characteristics of groups of participants with and without sensory loss using means and standard deviations or frequency distributions and compared groups of participants using t-tests for continuous variables, chi-square tests for categorical variables, and negative binomial regression for count variables.

Participants were grouped into 4 peripheral sensory loss categories based on their changes from baseline to 60 months: those with intact sensory function both at baseline and 60 months of follow-up (hereafter called **consistently intact**), those with intact sensory function at baseline and loss of function to at least mild impairment by 60 months (**progressing to impairment**), those with at least mildy impaired sensory function at both baseline and 60 months (**consistently impaired**), and those with impaired sensory function at baseline but intact function at 60 months. (**improved**) (Table 2). Those with mild-to-moderate and severe sensory loss were grouped together into the impaired category for the derivation of these sensory loss trajectories. Due to the small number of participants (9) in the improved category, this group was excluded from consideration in longitudinal analyses, leaving 342 participants for the analyses of change over time.

Analysis of the relationship between sensory loss categories and change in gait speed was done using repeated measures analysis of variance models. Gait speeds measured at baseline, 18 months, and 60 months were used as dependent variables, and tests of global mean differences across all time points as well as pairwise tests comparing mean gait speeds at each time point were conducted. The relationship to the falls rate outcome was analyzed using negative binomial regression, as falls rates exhibited a high degree of variance between participants through the course of study follow-up. Since falls were recorded as present or absent on each day of the 5-year followup period, there were no missing falls data.

Adjusted analyses for gait speed included age, sex, comorbidity count, use of a walking aid, baseline Berg balance score, and diabetes as covariates. Adjusted analyses for falls included age, sex, average weekly physical activity (PASE), baseline Berg balance score, diabetes, comorbidity count, and the SPPB score. For hypothesis testing, a two-sided type-I error probability of 0.05 was allowed. Models were estimated using Stata/MP version 13.1 (Statacorp, College Station, Tx).

## Results

The characteristics of the full study cohort and those with (mild, moderate, or severe) and without (intact) baseline sensory impairment are shown in Table 3. At baseline, participants with any sensory impairment were four years older on average than their intact counterparts, were more likely to be male, and had diminished executive function (as indicated by 20s longer Trails B time on average). They also exhibited more multimorbidity and diminished physical functioning than their intact counterparts, reporting a greater prevalence of diabetes and peripheral neuropathy, and 0.3 greater mean comorbidity count on average. They were twice as likely to report use of a walking aid and exhibited diminished physical function, scoring an average of 1.4 points lower on the SPPB and 0.1 m/s slower gait speed, both differences well above established thresholds of clinical significance. Despite these differences, however, a history of one or more falls was similar in the two groups.

**Table 3:** Baseline characteristics and descriptive statistics of the study sample (*N* = 351); mean (standard deviation) or count (percent) is shown

	Full cohort (*n* = 351)	Baseline somatosensory function	
		Intact (*n* = 292)	Impaired (*n* = 59)
Age, years	78 (5)	77 (5)	81 (5)
Female	230 (66%)	203 (70%)	27 (46%)
White	283 (81%)	230 (79%)	53 (90%)
Education			
Less than high school	19 (5%)	15 (5%)	4 (7%)
High school	68 (19%)	53 (18%)	15 (25%)
Some college/college	136 (39%)	119 (41%)	17 (29%)
Graduate/professional education	127 (36%)	104 (36%)	23 (39%)
Body mass index, kg/m^2^	27.2 (4.9)	27.0 (4.9)	27.9 (5.0)
Current smoker	11 (3%)	10 (3%)	1 (2%)
Daily alcohol use (percent yes)	34 (10%)	28 (10%)	6 (10%)
Comorbidity count^a^	2.4 (1.5)	2.4 (1.5)	2.7 (1.5)
CES-D score	9.9 (10.2)	9.8 (10.0)	10.3 (11.0)
Medication use			
Antihypertensive medication	237 (68%)	194 (66%)	43 (73%)
Antidepressants	35 (10%)	29 (10%)	6 (10%)
Anti-seizure medications	12 (3%)	10 (3%)	2 (4%)
Statins	160 (46%)	131 (45%)	29 (49%)
Anxiolytics	42 (12%)	33 (11%)	9 (15%)
Antihistamines	35 (10%)	29 (10%)	6 (10%)
Opioids	14 (4%)	10 (3%)	4 (7%)
Nonsteroidal anti-inflammatory drugs	65 (19%)	51 (18%)	14 (24%)
Analgesics/antipyretics	82 (24%)	71 (25%)	11 (19%)
Cognitive function			
Mini-Mental State Examination	27.7 (2.3)	27.8 (2.3)	27.5 (2.3)
Trail Making Test, seconds			
Part A	52 (33)	51 (33)	56 (33)
Part B	123 (68)	120 (66)	140 (72)
Part B less A	74 (55)	72 (55)	84 (55)
Medical conditions (self-report)			
Stroke	32 (9%)	26 (9%)	6 (10%)
Diabetes mellitus	47 (13%)	33 (11%)	14 (24%)
Hyperlipidemia	179 (51%)	148 (51%)	31 (53%)
Hypertension	259 (75%)	214 (74%)	45 (78%)
Peripheral artery disease	29 (8%)	22 (8%)	7 (12%)
History of back pain or spinal stenosis	138 (39%)	116 (40%)	22 (37%)
History of falls	129 (37%)	106 (36%)	23 (40%)
Parkinson’s disease	6 (2%)	5 (2%)	1 (2%)
Peripheral neuropathy	69 (21%)	35 (13%)	34 (63%)
Cancer, excluding skin cancer	81 (23%)	63 (22%)	18 (31%)
Physical function			
Uses walking aid	36 (10%)	24 (8%)	12 (20%)
Physical Activity Scale for the Elderly	111 (69)	114 (70)	97 (61)
Short Physical Performance Battery	9.8 (2.2)	10.0 (2.0)	8.6 (2.8)
Gait speed at baseline, m/s	1.00 (0.25)	1.02 (0.24)	0.92 (0.29)
Falls during first year of follow-up	1.1 (1.7)	1.1 (1.8)	1.0 (1.4)
Berg Balance score	51 (5)	52 (5)	48 (8)

^a^*Comorbidity count includes coronary heart disease, high blood pressure, diabetes, ulcer or other stomach disease, kidney disease, liver disease, anemia, cancer, depression, osteoarthritis and degenerative arthritis, rheumatoid arthritis, and other unlisted medical problem* [34]

**Fig. 1:**
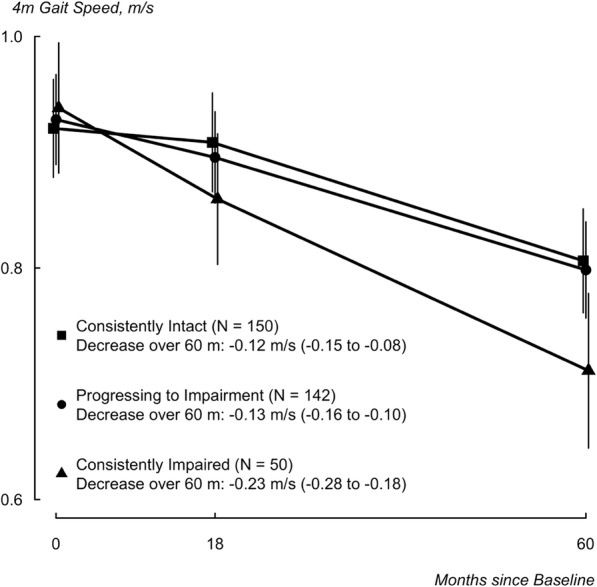
Model-generated mean and 95% confidence interval estimates of 60-month decrease in 4 m gait speed are provided for each temporal pattern of sensory functioning. Those ‘consistently intact’ displayed intact function at both baseline and follow-up measurements. Those ‘progressing to impairment’ exhibited intact perception at baseline but were impaired at follow-up. Those ‘consistently impaired’ had sensory impairment at both time points. Estimates and 95% confidence intervals were derived from repeated measures ANOVA adjusted for age, sex, comorbidity count, use of walking aids, baseline Berg balance score, and diabetes

The relationship between changes in peripheral sensory function and change in gait speed over 18 and 60 months is illustrated in Fig. 1. After model adjustment, participants in the three groups had comparable mean baseline gait speed. The group of subjects with consistently intact sensory function over this time period had the smallest declines in gait speed over 60 months (− 0.12 m/s; 95% CI: -0.15 to − 0.08). This decline in gait speed was of similar magnitude to the decline observed in subjects whose sensory function progressed to impairment (− 0.13 m/s; − 0.16 to − 0.10).Those whose sensory function was consistently impaired had a steeper decline in gait speed (− 0.23 m/s; 95% CI: -0.28 to − 0.18). The difference between the consistently impaired group and the others was statistically significant and consistent with a ‘substantially’ meaningful difference for gait speed metrics in older populations [36].

Declines in peripheral sensory function were also associated with fall risk. Table 4 shows the absolute and relative risk of falls by temporal pattern of impairment. Those whose sensory function progressed to impairment during followup had a greater risk of falls than those whose sensory function was consistently intact (adjusted risk ratio = 1.57 (95% confidence interval = 1.12 to 2.22). The group that remained consistently impaired over the 5 years had an elevated fall rate, but it was not statistically significantly different from that of the consistently intact group (adjusted risk ratio = 1.47, 95% confidence interval = 0.89 to 2.45).

**Table 4:** Risk of Falls According to temporal pattern in sensory function; point estimates and 95% confidence intervals shown

	Absolute Risk of Fall		^a^Relative Risk of Fall	
	Events (py)	Rate per py	Unadjusted	^b^Adjusted
Consistently intact	384 (474)	0.81	Referent	Referent
Progressing to impairment	591 (454)	1.31	1.60 (1.16 to 2.22)	1.57 (1.12 to 2.22)
Consistently impaired	181 (161)	1.12	1.39 (0.89 to 2.18)	1.48 (0.89 to 2.45)

*PY* = patient year

^a^Estimates obtained using negative binomial regression

^b^Adjusted for age, sex, physical activity (PASE index), diabetes, comorbidity, Short Physical Performance Battery score

## Discussion

The results of this study demonstrate the course of tactile sensory loss in the feet of older community-dwelling people over 5 years, and relationships between peripheral sensory loss and the concurrent slowing of gait and development of falls. Over the 5-year course of the study, older adults with consistently impaired peripheral sensory function had a significantly greater decline in gait speed compared to those with consistently intact or progressive impairment over 5 years. Compared to those with consistently intact sensory function, older adults who developed sensory impairments had a greater risk of falls. These results indicate that the loss of peripheral sensory function is a significant contributor to slowing of gait and an increased risk of falls in a community-dwelling older adult population.

The absolute number (59) and percent (17%) of participants with sensory impairment were relatively low in the MOBILIZE Boston population, but higher than the 7% reported in other older populations [11, 13]. Most of our participants had intact sensation at baseline, but this enabled us to observe the development of impairments over time. Nearly half (49%) of those initially intact developed sensory impairment over 5 years of followup. This high incidence of sensory loss has not been fully appreciated in other studies nor in clinical practice.

To our knowledge previous studies have not examined changes in peripheral sensory function over time nor their relationship to mobility outcomes. However, several cross-sectional and longitudinal studies have examined relationships between baseline abnormalities in foot sensation (including sense of vibration and touch), functional impairments, and fall risk. Most of these studies demonstrated that the loss of vibratory sensation was associated with increased fall risk [5, 37, 38]. For example, Patel et al. [37] found among older women with diabetes that fallers had higher vibratory sensory thresholds than non-fallers, but both fallers and non-fallers had similar touch sensation. Using cross-sectional data from the 2003–2004 National Health and Nutrition Examination Survey (NHANES), Wilson et al. [38] found no association between sensory loss in the feet, as measured using 10 g SWMT, and a subjective report of “difficulty with falls during the past 12 months,” assessed by questionnaire. However, after adjustments, participants who exhibited peripheral neuropathy had an increased risk of balance impairment compared to those without peripheral neuropathy. In a cross-sectional study exploring the effect of different sensory systems on postural stability, Lord and colleagues [5] found that poor tactile sensitivity to Semmes-Weinstein monofilaments at the lateral malleolus of the dominant ankle was associated with increased body sway. They also reported that peripheral sensation is the most important sensory system in the maintenance of static postural stability. Richardson et al. [39] found a cross-sectional relationship between peripheral neuropathy diagnosed by electromyography in a small referral population and a self-reported history of falls during the past year. Using longitudinal data from the Health, Aging and Body Composition study, Strotmeyer et al. [16] found that insensitivity to the 10 g Semmes Weinstein monofilament at the great toe was associated with lower quadriceps muscle strength. Thus, a number of neuromuscular deficits are associated with peripheral neuropathy and may lead to mobility impairments and falls.

Our longitudinal results are most consistent with those of Lord et al. [7] and Luukinen et al. [8] who found relationships between various modalities of sensory loss at the knees and recurrent falls in community-dwelling populations followed prospectively with fall questionnaires or diaries over 1 or 2 years, respectively. In Lord et al’s study, touch sensation did not discriminate non-fallers from single or recurrent fallers. However, in another one-year prospective study of older adults living in an intermediate care facility, Lord and Clark [40] found that tactile sensitivity using a Semmes Weinstein Pressure Aesthesiometer did discriminate between fallers and non-fallers.

The subjects of our study who exhibited consistent sensory impairment at baseline and at follow-up were not more likely to fall than those who were consistently intact. The lack of a statistically significant difference between these groups could be due to the relatively small size of the consistently impaired group (*N* = 50) or adaptive behaviors learned by people with chronic sensory loss. Those with baseline sensory impairment were more likely to be using walking aids, which may have protected them from falls. There was also a trend towards lower physical activity as measured by PASE, suggesting that this group may have had less time at risk for falls.

The strength of the current study lies in its 5-year longitudinal design and rigorous detection of falls using state-of-the-art falls calendars and follow-up phone calls. There are several weaknesses to the current study. Participants did not receive a full neuropathic evaluation and the monofilament assessment of sensory function is only a quick screen that is specific, but not very sensitive to neuropathic sensory loss [41]. However, this simple, widely available, bed-side test of sensation on the dorsum of the great toe was sensitive to changes over time and predictive of a decrease in gait speed and increase in fall rate, even in the absence of more rigorous assessments of vibratory, proprioceptive, and motor nerve function. Although observed relationships could be confounded by underlying diseases such as diabetes, which affect sensation, gait and falls, the findings persisted after multivariate adjustments.

Our longitudinal results provide validation of the belief that peripheral sensory loss is a risk factor for mobility impairment. They also suggest that a decline in touch sensation at the great toe over time may be a more important predictor of slow gait speed and falls than a baseline sensory deficit. Therefore, simple, repeated clinical assessments of sensory function in the feet of older adults may be helpful in identifying and treating those at risk of falls to prevent their morbid consequences.

## Abbreviations

BMI: Body mass index
MOBILIZE: Maintenance Of Balance, Independent Living, Intellect, and Zest in the Elderly
PASE: Physical Activity Scale for the Elderly
SPPB: Short Physical Performance Battery
SWMT: Semmes-Weinstein monofilament test

## References

[1] Centers for Disease Control and Prevention. Falls are Leading Cause of Injury and Death in Older Americans. CDC Newsroom. 2016. https://www.cdc.gov/media/releases/2016/p0922-older-adult-falls.html. Accessed 22 Sept 2016.

[2] Gu Y, Dennis SM (2017). Are falls prevention programs effective at reducing the risk factors for falls in people with type-2 diabetes mellitus and peripheral neuropathy: a systematic review with narrative synthesis. J Diabetes Complicat.

[3] Rubenstein LZ (2006). Falls in older people: epidemiology, risk factors and strategies for prevention. Age Ageing.

[4] Richardson JK, Hurvitz EA (1995). Peripheral neuropathy: a true risk factor for falls. J Gerontol A Biol Sci Med Sci.

[5] Lord SR, Clark RD, Webster IW (1991). Physiological factors associated with falls in an elderly population. J Am Geriatr Soc.

[6] Lord SR, Clark RD, Webster IW (1991). Postural stability and associated physiological factors in a population of aged persons. J Gerontol.

[7] Lord SR, Ward JA, Williams P, Anstey K (1994). Physiological factors associated with falls in older community-dwelling women. J Am Geriatr Soc.

[8] Luukinen H, Koski K, Laippala P, Kivela SL (1995). Predictors for recurrent falls among the home-dwelling elderly. Scand J Prim Health.

[9] Cavanagh PR, Derr JA, Ulbrecht JS, Maser RE, Orchard TJ (1992). Problems with gait and posture in neuropathic patients with insulin-dependent diabetes mellitus. Diabetic Med.

[10] Baldereschi M, Inzitari M, Di Carlo A (2007). Epidemiology of distal symmetrical neuropathies in the Italian elderly. Neurology.

[11] Chiles NS, Phillips CL, Volpato S (2014). Diabetes, peripheral neuropathy, and lower-extremity function. J Diabetes Complicat.

[12] Katon JG, Reiber GE, Nelson KM (2013). Peripheral neuropathy defined by monofilament insensitivity and diabetes status. Diabetes Care.

[13] Hanewinckel R, Van Oijen M, Ikram MA, Van Doorn PA (2016). The epidemiology and risk factors of chronic polyneuropathy. Eur J Epidemiol.

[14] Callaghan B, Kerber K, Langa KM (2015). Longitudinal patient-oriented outcomes in neuropathy: importance of early detection and falls. Neurology.

[15] Dyck PJ, Litchy WJ, Lehman KA, Hokanson JL, Low PA, O'Brien PC (1995). Variables influencing neuropathic endpoints: the Rochester diabetic neuropathy study of healthy subjects. Neurology.

[16] Strotmeyer ES, De Rekeneire N, Schwartz AV (2009). Sensory and motor peripheral nerve function and lower-extremity quadriceps strength: the health, aging and body composition study. J Am Geriatr Soc.

[17] Wuehr M, Schniepp R, Schlick C (2014). Sensory loss and walking speed related factors for gait alterations in patients with peripheral neuropathy. Gait Posture.

[18] Leveille SG, Kiel DP, Jones RN (2008). The MOBILIZE Boston study: design and methods of a prospective cohort study of novel risk factors for falls in an older population. BMC Geriatr.

[19] Samelson EJ, Kelsey JL, Kiel DP (2008). Issues in conducting epidemiologic research among elders: lessons from the MOBILIZE Boston study. Am J Epidemiol.

[20] Folstein MF, Folstein SE, McHugh PR (1975). “Mini-mental state”. A practical method for grading the cognitive state of patients for the clinician. J Psychiatr Res.

[21] Escobar JI, Burnam A, Karno M, Forsythe A, Landsverk J, Golding JM (1986). Use of the mini-mental state examination (MMSE) in a community population of mixed ethnicity cultural and linguistic artifacts. J Nerv Ment Dis.

[22] Tombaugh TN (2004). Trail making test a and B: normative data stratified by age and education. Arch Clin Neuropsychol.

[23] Guralnik JM, Simonsick EM, Ferrucci L (1994). A short physical performance battery assessing lower extremity function: association with self-reported disability and prediction of mortality and nursing home admission. J Gerontol.

[24] Berg KO, Maki BE, Williams JI, Holliday PJ, Wood-Dauphinee SL (1992). Clinical and laboratory measures of postural balance in an elderly population. Arch Phys Med Rehabil.

[25] Perkins BA, Olaleye D, Zinman B, Bril V (2001). Simple screening tests for peripheral neuropathy in the diabetes clinic. Diabetes Care.

[26] Abbott CA, Carrington AL, Ashe H (2002). The north-west diabetes foot care study: incidence of, and risk factors for, new diabetic foot ulceration in a community-based patient cohort. Diabet Med.

[27] Dros J, Wewerinke A, Bindels PJ, Van Weert HC (2009). Accuracy of monofilament testing to diagnose peripheral neuropathy: a systematic review. Ann Fam Med.

[28] Shumway-Cook A, Baldwin M, Polissar NL, Gruber W (1997). Predicting the probability for falls in community-dwelling older adults. Phys Ther.

[29] Guralnik JM, Ferrucci L, Simonsick EM, Salive ME, Wallace RB (1995). Lower-extremity function in persons over the age of 70 years as a predictor of subsequent disability. N Engl J Med.

[30] Guralnik JM, Ferrucci L, Pieper CF (2000). Lower extremity function and subsequent disability: consistency across studies, predictive models, and value of gait speed alone compared with the short physical performance battery. J Gerontol A Biol Sci Med Sci.

[31] Gibson MJ, Andres RO, Isaacs B, Radebaugh T, Wormpetersen J (1987). The prevention of falls in later life - a report of the Kellogg-international-work-group on the prevention of falls by the elderly. Dan Med Bull.

[32] Tinetti ME, Liu WL, Claus EB (1993). Predictors and prognosis of inability to get up after falls among elderly persons. JAMA.

[33] Washburn RA, Smith KW, Jette AM, Janney CA (1993). The physical activity scale for the elderly (PASE): development and evaluation. J Clin Epidemiol.

[34] Sangha O, Stucki G, Liang MH, Fossel AH, Katz JN (2003). The self-administered comorbidity questionnaire: a new method to assess comorbidity for clinical and health services research. Arthritis Rheum.

[35] Eaton WW, Muntaner C, Smith C, Tien A, Ybarra M, ME M (2004). Center for Epidemiologic Studies Depression Scale: review and revision (CESD and CESD-R). The use of psychological testing for treatment planning and outcomes assessment.

[36] Perera S, Mody SH, Woodman RC, Studenski SA (2006). Meaningful change and responsiveness in common physical performance measures in older adults. J Am Geriatr Soc.

[37] Patel S, Hyer S, Tweed K (2008). Risk factors for fractures and falls in older women with type 2 diabetes mellitus. Calcified Tissue Int.

[38] Wilson SJ, Garner JC, Loprinzi PD (2016). The influence of multiple sensory impairments on functional balance and difficulty with falls among U.S. adults. Prev Med.

[39] Richardson JK, Ching C, Hurvitz EA (1992). The relationship between electromyographically documented peripheral neuropathy and falls. J Am Geriatr Soc.

[40] Lord SR, Clark RD (1996). Simple physiological and clinical tests for the accurate prediction of falling in older people. Gerontology.

[41] Feng Y, Schlösser FJ, Sumpio BE (2009). The Semmes Weinstein monofilament examination as a screening tool for diabetic peripheral neuropathy. J Vasc Surg.

